# Urinary Bisphenol A Levels during Pregnancy and Risk of Preterm Birth

**DOI:** 10.1289/ehp.1408126

**Published:** 2015-03-27

**Authors:** David E. Cantonwine, Kelly K. Ferguson, Bhramar Mukherjee, Thomas F. McElrath, John D. Meeker

**Affiliations:** 1 Division of Maternal-Fetal Medicine, Department of Obstetrics and Gynecology, Brigham and Women’s Hospital, Harvard Medical School, Boston, Massachusetts, USA; 2 Department of Environmental Health Sciences, and; 3 Department of Biostatistics, University of Michigan School of Public Health, Ann Arbor, Michigan USA

## Abstract

**Background:**

Preterm birth (PTB), a leading cause of infant mortality and morbidity, has a complex etiology with a multitude of interacting causes and risk factors. The role of environmental contaminants, particularly bisphenol A (BPA), is understudied with regard to PTB.

**Objectives:**

In the present study we examined the relationship between longitudinally measured BPA exposure during gestation and PTB.

**Methods:**

A nested case–control study was performed from women enrolled in a prospective birth cohort study at Brigham and Women’s Hospital in Boston, Massachusetts, during 2006–2008. Urine samples were analyzed for BPA concentrations at a minimum of three time points during pregnancy on 130 cases of PTB and 352 randomly assigned controls. Clinical classifications of PTB were defined as “spontaneous,” which was preceded by spontaneous preterm labor or preterm premature rupture of membranes, or “placental,” which was preceded by preeclampsia or intrauterine growth restriction.

**Results:**

Geometric mean concentrations of BPA did not differ significantly between cases and controls. In adjusted models, urinary BPA averaged across pregnancy was not significantly associated with PTB. When examining clinical classifications of PTB, urinary BPA late in pregnancy was significantly associated with increased odds of delivering a spontaneous PTB. After stratification on infant’s sex, averaged BPA exposure during pregnancy was associated with significantly increased odds of being delivered preterm among females, but not males.

**Conclusions:**

These results provide little evidence of a relationship between BPA and prematurity, though further research may be warranted given the generalizability of participant recruitment from a tertiary teaching hospital, limited sample size, and significant associations among females and within the clinical subcategories of PTB.

**Citation:**

Cantonwine DE, Ferguson KK, Mukherjee B, McElrath TF, Meeker JD. 2015. Urinary bisphenol A levels during pregnancy and risk of preterm birth. Environ Health Perspect 123:895–901; http://dx.doi.org/10.1289/ehp.1408126

## Introduction

Preterm birth (PTB), defined as delivery before 37 weeks completed gestation, is a leading cause of infant mortality and significant precursor to future morbidity. Overall rates of PTB in the United States are significantly higher than several decades ago, with persistent racial and economic disparity [Institute of Medicine (IOM) 2006]. The etiology of preterm birth is recognized to have a multitude of overlapping factors, many of which are understudied, leading to the inability of clinicians and health care providers to provide good prevention and/or treatment options. One critical area that is understudied with regard to potential contribution to the etiology of PTB is environmental contaminant exposure. Many environmental exposures are modifiable through behavioral change and thus represent an attractive target for prevention.

Bisphenol A (BPA) is a high production–volume chemical most commonly used in the manufacturing of epoxy resins and polycarbonate polymers. Downstream applications include, but are not limited to, a variety of consumer products such as food can linings, water bottles, dental sealants, thermal receipts, medical equipment, flooring, reusable food and drink containers, and water supply pipes. As these products age or are exposed to high heat or acidic/basic conditions, monomers can be released to the environment (Arnold 1996; Brede et al. 2003; Nerín et al. 2003). Because of their widespread use and leaching from consumer products, it is not surprising that detectable urinary BPA concentrations have been found in various populations, including pregnant women (Calafat et al. 2008; He et al. 2009; Meeker et al. 2013; Vandenberg et al. 2012; Ye et al. 2008). BPA has also been detected in the serum of pregnant women, follicular fluid, placental tissue and cord blood; but of particular concern, due to the sensitive developmental period for fetuses, is evidence of higher amniotic fluid BPA concentrations in early compared with late pregnancy (Edlow et al. 2012; Ikezuki et al. 2002; Lee et al. 2008; Philippat et al. 2013; Yamada et al. 2002). Of particular note, it has been shown that even during pregnancy urinary BPA levels can widely vary, with several studies indicating weak intraclass correlation coefficients (ICCs), suggesting the importance of taking multiple measurements to gain a greater understanding of the variance in exposure during this critical developmental period (Braun et al. 2011a, 2012; Meeker et al. 2013).

Toxicological evidence suggests that BPA exposure may affect pregnancy through a variety of hormone-mediated mechanisms. Initially considered to be a weak environmental estrogen, BPA more recently in experimental models has been shown to stimulate biological responses at very low concentrations and has been demonstrated to be as potent as estradiol (E_2_) in some of its effects (Alonso-Magdalena et al. 2005, 2008; Hugo et al. 2008; Zsarnovszky et al. 2005). Additionally, there is evidence that BPA can also alter thyroid signaling, bind to the glucocorticoid receptor, act as an anti-androgen, and trigger activation of a variety of signal transduction pathways affecting cell proliferation, apoptosis, and survival (Kaneko et al. 2008; Steinmetz et al. 1997; Wetherill et al. 2007; Zoeller 2007). For example, it has been demonstrated that BPA can affect the proliferative process of trophoblastic cells through estrogen-related receptor-γ (ERRγ1) (Morice et al. 2011) and has a dose-dependent effect upon apoptosis of primary human cytotrophoblast cells via tumor necrosis factor-α (Benachour and Aris 2009). These results imply a direct impact on placental function, which if perturbed can alter the normal course of pregnancy.

Current epidemiological evidence for the association of BPA exposure with adverse birth outcomes, specifically PTB, are extremely limited (Cantonwine et al. 2010; Padmanabhan et al. 2008; Weinberger et al. 2014; Wolff et al. 2008). In a small nested case–control study (*n* = 60) of PTB in Mexico City, researchers found that the adjusted odds ratio of delivering at < 37 weeks in relation to a 1-log increase in specific gravity–adjusted third-trimester BPA concentration was 2.5 [95% confidence interval (CI): 1.1, 6.0] (Cantonwine et al. 2010). Several other studies assessing gestational age as a continuous variable have found inconsistent results (Padmanabhan et al. 2008; Weinberger et al. 2014; Wolff et al. 2008). All of the above studies failed to account for the high variability in BPA exposure across pregnancy, using a single spot urine sample for exposure assessment, and the heterogeneous etiologies of preterm birth (IOM 2006; McElrath et al. 2008).

In the present study we examined the relationship between longitudinally measured urinary BPA concentrations during gestation and preterm birth. We further assessed the associations between gestational BPA exposure and more specific classifications of PTB.

## Methods

*Study population*. The parent birth cohort consisted of a total of 2,246 women, 18–50 years of age, who were recruited at three tertiary care academic centers: Brigham and Women’s Hospital and Beth Israel Deaconess Medical Center in Boston, Massachusetts, and The Hospital of the University of Pennsylvania in Philadelphia, Pennsylvania, between October 2006 and September 2008. Eligibility included those women who sought out routine prenatal care before 15 weeks completed gestation, who were > 18 years of age, and planned to deliver at the enrolling institutes. Exclusion criteria included higher-order multiple gestations (triplets or greater). Written informed consent was obtained from all participating women, and the study protocol was approved by each institutional review board. In 2011, a nested case–control study of singleton PTBs was selected from the Brigham and Women’s Hospital participant pool (*n* = 1,648), who were originally enrolled as part of this larger prospective birth cohort. These women were originally recruited at two Brigham and Women’s Hospital clinical facilities and one private practice facility. This nested case–control study consisted of 130 women who delivered before 37 weeks of gestation and 352 randomly selected women who delivered at or after 37 weeks. The study was approved by the institutional review boards of Brigham and Women’s Hospital and the University of Michigan.

Maternal urine samples were obtained at four visits during pregnancy. Initial visit samples were collected at median 9.7 weeks gestation (range, 4.7–16.1 weeks), visit 2 at median 17.9 weeks (range, 14.9–21.9 weeks), visit 3 at median 26.0 weeks (range, 22.9–29.3 weeks), and visit 4 at median 35.1 weeks (range, 33.1–38.3 weeks). All specimens were stored at –80°C until analysis. Demographic information was collected at the initial visit. Clinically relevant pregnancy characteristics were collected at the initial visit and subsequently at three additional time points throughout pregnancy. Gestational age was confirmed by ultrasound scanning at < 15 weeks gestation if inconsistent with last menstrual period dating.

We further classified preterm birth by clinical presentation (McElrath et al. 2008). In this analysis there were 56 “spontaneous” PTB [arising from clinical presentation of spontaneous preterm labor and/or preterm premature rupture of membranes (PPROM)] and 35 “placental” preterm births (comprising PTB following preeclampsia or intrauterine growth restriction). Additionally, 39 cases were excluded from this subset analysis because they were delivered because medical protocol required their elective delivery before 37 weeks. The deliveries that were performed due to obstetrical protocol included the management of prior classical cesarean section, abdominal cerclage, prior term intrauterine demise, and suspected uterine wall thinning (due to prior surgery). These cases were not analyzed separately, as they have no known unifying etiology.

*Urinary BPA concentrations*. Total BPA (free + conjugated) was measured in all available urine samples (*n* = 1,695) by NSF International in Ann Arbor, Michigan, based on methods developed by the Centers for Disease Control and Prevention (CDC) (Lewis et al. 2013; Silva et al. 2007). Levels below the limit of detection (LOD) were kept if a numerical value was reported or replaced by dividing the LOD (0.4 ng/mL) by the square root of two if no value was reported (Hornung and Reed 1990). Urinary specific gravity (SG) was measured in all samples as an indicator of urine dilution using a digital handheld refractrometer (ATAGO Company Ltd., Tokyo, Japan). Urinary BPA concentrations were corrected for SG using the following formula: *P_c_ = P*[(1.015 – 1)/(*SG* – 1)], where *P_c_* represents the SG-corrected BPA concentration (nanograms per milliliter), *P* represents the measured concentration in urine, 1.015 is the median SG of all samples measured, and *SG* represents the specific gravity of the individual sample (Meeker et al. 2009). Both uncorrected and SG-corrected metabolite levels were log-normally distributed and were ln-transformed for statistical analysis to more closely approximate normality and to reduce the likelihood of influential values given the skewed distribution.

*Statistical analysis*. Analysis was performed using SAS version 9.2 (SAS Institute Inc., Cary, NC) and R version 2.15.2 (R Core Team 2014). *p*-Values < 0.05 were used to define statistical significance. Sociodemographic characteristics of the participating women have been previously described (Ferguson et al. 2014), and associations with cases/control status were examined using chi-square tests. To assess variability in BPA levels across pregnancy, we examined differences in levels by visit both in the population overall and in cases and controls separately and tested the differences by Wilcoxon rank sum test. Geometric means and standard deviations of SG-corrected BPA levels at individual visits were calculated and differences by visit were tested using linear mixed models with random intercepts for subject to adjust for intraindividual correlation. Spearman correlations between measures of BPA across subjects were calculated using SG-corrected values. To examine temporal variability in BPA levels by subject, ICCs and 95% CIs were calculated using uncorrected and SG-corrected BPA.

Geometric average BPA concentrations were calculated using the visit 1–visit 3 time point measurements. Visit 4 measurements were excluded from the average because of a relatively small proportion of cases with samples available from that time point. Crude logistic regression models, where preterm birth was the outcome, included average urinary specific gravity and BPA concentrations. In full models, maternal age, race/ethnicity (white/African American/other), and education level were included *a priori*, and additional covariates were added in a forward step-wise model selection procedure with inclusion in final models if they altered log-transformed BPA concentration effect estimates by > 10%. Additional variables that were considered included health insurance category (private/HMO/self-pay vs. Medicaid/SSI/MassHealth), prepregnancy body mass index (BMI), smoking status during pregnancy (yes/no), parity (nulliparous/parous), prior history of preterm birth (yes/no), and use of assisted reproductive technology (ART) (yes/no). As a sensitivity analysis, we explored whether addition of either time point specific summed di(2-ethyheyxl) phthalate (ΣDEHP) or average urinary ΣDEHP, which were shown earlier in this population to be associated with an increased odds of PTB, altered any effect estimates (Ferguson et al. 2014). Adjustment for mono-*n*-butyl phthalate (MBP), mono(2-ethylhexyl) phthalate (MEHP), and mono(2-ethyl-5-carboxypentyl) phthalate (MECPP) was also explored, but results were not included because separate adjustment did not alter the results seen with adjustment for ΣDEHP. ΣDEHP, MEHP, MECPP, and MBP metabolites were associated with preterm birth in a previous study (Ferguson et al. 2014) in this population, and levels were weakly but significantly correlated with BPA in this study (Spearman rho = 0.17, 0.11, 0.12, and 0.28, respectively). Phthalate measurement methods and primary associations with PTB are provided in detail by Ferguson et al. (2014).

Windows of vulnerability to BPA exposure were then assessed by fitting separate logistic regression models with preterm birth as the outcome to calculate odds ratios (ORs) corresponding to BPA levels from each individual visit and, due to low correlation between time points, together in the same model. We did not control for multiple comparisons.

To explore the longitudinal nature of the relationship between BPA exposure and risk of PTB, we initially used linear mixed-effects models to generate random slopes and intercepts for BPA exposure over time. Random intercepts and slopes were then used as predictors in the adjusted logistic regression models. We also fit generalized additive mixed models (GAMMs) and linear mixed-effects models using BPA concentrations as the response variable, and preterm birth, gestational age at sample collection, and other covariates as the explanatory variables, to explore the interaction between preterm birth and gestational age at time of urine sample collection.

For additional secondary analyses, we repeated the above steps for subtypes of preterm birth, including placental and spontaneous preterm birth. PTB cases that did not fit into the subtype were excluded from analysis instead of being recoded as controls. We additionally recreated models of overall preterm birth stratified by infant sex. Covariates from full models were kept the same for sensitivity analyses for comparison.

## Results

Study demographic characteristics have been previously described (Ferguson et al. 2014). Briefly, the study population was predominantly white (58.5%) and highly educated (85.9% had some college-level education) and were nonsmokers (92.3%). Approximately 10% of the women used some form of ART, and 47.3% of the population was either overweight (25 to ≤ 30 kg/m^2^ BMI) or obese (> 30 kg/m^2^ BMI). These population characteristics did not differ significantly between PTB cases and controls (Ferguson et al. 2014). Overall, BPA concentrations were detected in 1,350 (79.6%) of the samples analyzed, with the breakdown by visit and case–control status detailed in Table 1. There were 327 (92.9%) controls and 114 (87.7%) cases that had three or more repeated samples available for analysis during pregnancy. Visit 4 urine samples were collected on 66 (50.8%) cases and 314 (89.2%) controls. The mean (range) of the final gestational age for those cases who provided a visit 4 urine sample was 36.3 (35.0–36.9) weeks.

**Table 1:** Specific gravity–corrected BPA concentration (ng/mL) geometric means (GMs) and standard deviations (GSDs) by visit.

Outcome group	Visit 1		Visit 2		Visit 3		Visit 4	
	*n* (% > LOD)	GM (GSD)	*n* (% > LOD)	GM (GSD)	*n* (% > LOD)	GM (GSD)	*n* (% > LOD)	GM (GSD)
Overall	481 (81.9)	1.37 (2.32)	422 (73.7)	1.32 (2.18)	412 (81.5)	1.38 (2.32)	380 (81.6)	1.34 (2.24)
Cases	130 (80.8)	1.52 (2.48)	118 (70.3)	1.45 (2.32)	111 (82.9)	1.37 (2.53)	66 (86.4)	1.50 (2.64)
Spontaneous	56 (82.1)	1.52 (2.28)	52 (78.8)	1.40 (2.17)	47 (85.1)	1.31 (2.30)	25 (92.0)	2.24 (3.20)
Placental	35 (82.9)	1.61 (2.66)	31 (61.3)	1.64 (2.97)	32 (81.2)	1.50 (3.03)	15 (80.0)	1.48 (2.42)
Controls	351 (82.3)	1.31 (2.25)	304 (75.0)	1.27 (2.12)	301 (81.1)	1.39 (2.25)	314 (80.6)	1.31 (2.15)

As expected, BPA was log-normally distributed and levels were ln-transformed for statistical analysis. SG-corrected urinary BPA geometric means and standard deviations by individual visit are presented in Table 1. Concentrations were relatively constant across gestation, with no significant BPA concentration differences at individual visits by Wilcoxon rank-sum test between PTB cases and controls. Urinary specific gravity was the highest early in pregnancy (SG = 1.017) and had a significantly decreased trend across pregnancy (*p* < 0.01). Spearman correlations between study visits for BPA were low and ranged from 0.17 to 0.26 (all *p*-values < 0.01). ICC for SG-corrected BPA indicated low temporal reliability (ICC = 0.21; 95% CI: 0.16, 0.27), and ICCs were slightly higher in cases of PTB compared with controls (Table 2). SG concentrations had low to moderate reproducibility across pregnancy (ICC = 0.38; 95% CI: 0.33, 0.43).

**Table 2:** BPA intraclass correlation coefficients (95% CIs) in total population and by case status, both uncorrected and corrected for urinary specific gravity.

Analyte	Uncorrected			SG-corrected		
	Overall	Cases	Controls	Overall	Cases	Controls
BPA	0.32 (0.27, 0.38)	0.33 (0.23, 0.44)	0.32 (0.26, 0.38)	0.21 (0.16, 0.27)	0.25 (0.16, 0.37)	0.19 (0.14, 0.26)
SG	0.38 (0.33, 0.43)	0.39 (0.29, 0.50)	0.38 (0.32, 0.43)

Crude and adjusted ORs (95% CIs) of overall preterm birth in association with averaged and cross-sectional (i.e., by visit) urinary BPA levels are presented in Table 3. Adjusted models controlled for maternal age, maternal race/ethnicity, educational level attained, medical insurance, parity, prior history of preterm birth, and BMI. A third set of models additionally included subject-specific averages for the ΣDEHP metabolites.

**Table 3:** Odds ratios (95% CIs) of overall preterm birth in association with ln-unit increase in BPA concentration (ng/mL).

Overall preterm	Model 1^*a*^			Model 2^*b*^			Model 3^*c*^		
	*n* (cases, controls)	OR (95% CI)	*p*-Value	*n* (cases, controls)	OR (95% CI)	*p*-Value	*n* (cases, controls)	OR (95% CI)	*p*-Value
Geometric average (visit 1–3)	130, 352	1.19 (0.87, 1.65)	0.28	127, 336	1.21 (0.79, 1.85)	0.38	127, 336	1.15 (0.75, 1.77)	0.53
Visit 1	130, 351	1.21 (0.95, 1.54)	0.12	127, 333	1.11 (0.84, 1.45)	0.47	127, 333	1.08 (0.82, 1.42)	0.61
Visit 2	118, 334	1.19 (0.91, 1.57)	0.21	115, 293	1.25 (0.92, 1.70)	0.16	115, 293	1.24 (0.90, 1.71)	0.18
Visit 3	111, 301	0.95 (0.73, 1.23)	0.68	108, 287	0.92 (0.69, 1.23)	0.55	108, 287	0.85 (0.63, 1.16)	0.44
Visit 4	66, 314	1.10 (0.78, 1.53)	0.59	64, 298	1.29 (0.88, 1.87)	0.19	64, 298	1.28 (0.87, 1.90)	0.22
Combined model (visit 1–3)^*d*^	102, 263			99, 254			99, 254
Visit 1		1.24 (0.92, 1.68)	0.16		1.18 (0.85, 1.65)	0.32		1.18 (0.84, 1.64)	0.34
Visit 2		1.22 (0.90, 1.67)	0.21		1.34 (0.95, 1.89)	0.10		1.30 (0.91, 1.84)	0.15
Visit 3		0.88 (0.66, 1.17)	0.38		0.85 (0.62, 1.16)	0.30		0.82 (0.60, 1.12)	0.21
^***a***^Adjusted for specific gravity. ^***b***^Model adjusted for specific gravity, maternal age, race, maternal educational level attained, medical insurance, parity, prior history of PTB, and BMI. ^***c***^Additional adjustment for geometric average sum of DEHP metabolites. ^***d***^Combined model adjusted for all three study visits simultaneously.									

Generally, in both crude and adjusted models no significant relationships were observed between overall preterm birth and either averaged (OR = 1.21; 95% CI: 0.79, 1.85) or individual-visit urinary BPA levels, although, except for visit 3, ORs tended to be > 1.0 (Table 3). In our joint models using the random slopes and intercepts as predictors in the adjusted logistic regression models, we found no significant associations (see Supplemental Material, Table S1). Fitting generalized additive mixed models indicated that the association between gestational age at sample collection and urinary BPA concentrations was linear, and there was no interaction with PTB (data not shown). Follow-up analysis using linear mixed-effects models confirmed no significant interactions between preterm birth and gestational age at urine sampling (interaction term: B = –0.00198; SE = 0.005; *p* = 0.67). Table 4 presents the secondary analysis in which PTB was further classified into either spontaneous or placental. We observed no significantly elevated odds for placental PTB in relation to averaged or visit-specific BPA concentrations. It is worth noting, though, that the ORs for the placental subtype PTB are consistently positive, with a suggestive increase in odds in relation to visit 2 BPA concentrations, and should be followed up in a larger study. Except for visit 4, there were no significant associations between BPA levels and spontaneous PTB. At this time point, a ln-unit increase in BPA concentrations was associated with 2.46 (95% CI: 1.42, 4.25) times greater odds of delivering a spontaneous PTB (*n* = 25). This relationship persisted even after additional adjustment for ΣDEHP metabolites. No significant relationships were observed between BPA concentrations and the medical protocol–driven PTBs (data not shown).

**Table 4:** Odds ratios (95% CIs) of preterm birth subcategories in association with ln-unit increase in BPA concentration (ng/mL).

Preterm subcategory	Model 1^*a*^			Model 2^*b*^			Model 3^*c*^		
	*n* (cases, controls)	OR (95% CI)	*p*-Value	*n* (cases, controls)	OR (95% CI)	*p*-Value	*n* (cases, controls)	OR (95% CI)	*p*-Value
Placental PTB
Average(visit 1–3)	35, 352	1.60 (0.91, 2.83)	0.10	33, 334	1.56 (0.75, 3.27)	0.24	33, 334	1.42 (0.66, 3.06)	0.37
Visit 1	35, 351	1.35 (0.92, 1.98)	0.13	33, 333	1.26 (0.77, 2.04)	0.36	33, 333	1.17 (0.71, 1.94)	0.54
Visit 2	31, 304	1.40 (0.91, 2.18)	0.13	29, 293	1.66 (0.93, 2.95)	0.08	29, 293	1.66 (0.90, 3.06)	0.10
Visit 3	32, 301	1.02 (0.53, 1.55)	0.93	30, 287	1.12 (0.66, 1.91)	0.67	30, 287	1.06 (0.60, 1.87)	0.83
Visit 4	15, 314	1.06 (0.54, 2.10)	0.87	13, 298	1.24 (0.53, 2.92)	0.62	13, 298	1.11 (0.45, 2.75)	0.81
Combined model (visit 1–3)^*d*^	29, 263			27, 254			27, 254
Visit 1		1.12 (0.68, 1.86)	0.65		0.99 (0.50, 1.94)	0.97		0.98 (0.50, 1.93)	0.96
Visit 2		1.45 (0.88, 2.37)	0.14		2.00 (1.00, 4.00)	0.05		1.93 (0.94, 3.94)	0.07
Visit 3		0.90 (0.57, 1.42)	0.66		0.80 (0.43, 1.49)	0.48		0.77 (0.40, 1.48)	0.44
Spontaneous PTB
Average(visit 1–3)	56, 352	1.42 (0.88, 2.29)	0.15	56, 334	1.32 (0.77, 2.26)	0.31	56, 334	1.19 (0.68, 2.06)	0.55
Visit 1	56, 351	1.15 (0.83, 1.59)	0.40	56, 333	1.06 (0.75, 1.50)	0.74	56, 333	1.01 (0.71, 1.44)	0.96
Visit 2	52, 304	1.18 (0.82, 1.70)	0.36	52, 293	1.19 (0.80, 1.77)	0.39	52, 293	1.20 (0.80, 1.80)	0.38
Visit 3	47, 301	0.90 (0.63, 1.30)	0.58	47, 287	0.79 (0.54, 1.18)	0.25	47, 287	0.68 (0.44, 1.05)	0.08
Visit 4	25, 314	1.86 (1.18, 2.94)	0.01	25, 298	2.46 (1.42, 4.25)	0.001	25, 298	2.25 (1.28, 4.00)	0.005
Combined model (visit 1–3)^*d*^	44, 254			44, 254			44, 254
Visit 1		1.13 (0.75, 1.71)	0.55		1.09 (0.71, 1.70)	0.69		1.08 (0.69, 1.68)	0.75
Visit 2		1.17 (0.77, 1.78)	0.46		1.27 (0.81, 2.00)	0.30		1.24 (0.79, 1.96)	0.35
Visit 3		0.90 (0.62, 1.32)	0.59		0.82 (0.55, 1.22)	0.33		0.78 (0.51, 1.18)	0.23
Placental PTB includes preterm births arising from preeclampsia or intrauterine growth restriction. Spontaneous PTB includes preterm births arising from preterm labor, premature rupture of membranes, or cervical insufficiency.^***a***^Adjusted for specific gravity. ^***b***^Model adjusted for specific gravity, maternal age, race, maternal educational level attained, medical insurance, parity, prior history of PTB, and BMI. ^***c***^Additional adjustment for geometric average sum of DEHP metabolites. ^***d***^Combined model adjusted for all three study visits simultaneously.									

After stratifying by sex, we observed significantly elevated odds of overall PTB for female infants in relation to averaged BPA concentrations (OR = 1.80; 95% CI: 1.02, 3.13), though statistical significance did not persist after additional adjustment for phthalate concentrations (Table 5). We observed no significant associations between average or visit-specific BPA exposure and PTB for male infants (OR = 0.89; 95% CI: 0.47, 1.70).

**Table 5:** Odds ratios (95% CIs) of overall preterm birth in association with ln-unit increase in BPA concentration (ng/mL) stratified by infant sex.

Overall preterm	Model 1^*a*^			Model 2^*b*^			Model 3^*c*^		
	*n* (cases, controls)	OR (95% CI)	*p*-Value	*n* (cases, controls)	OR (95% CI)	*p*-Value	*n* (cases, controls)	OR (95% CI)	*p*-Value
Female
Geometric average (visit 1–3)	56, 158	1.62 (0.98, 2.67)	0.06	55, 153	1.80 (1.02, 3.13)	0.04	55, 153	1.59 (0.89, 2.75)	0.15
*Visit 1*	56, 158	1.42 (1.02, 1.98)	0.04	55, 153	1.34 (0.89, 2.02)	0.17	55, 153	1.19 (0.78, 1.82)	0.41
*Visit 2*	50, 138	1.31 (0.89, 1.93)	0.17	49, 136	1.43 (0.93, 2.19)	0.10	49, 136	1.38 (0.88, 2.16)	0.16
*Visit 3*	45, 140	1.08 (0.75, 1.57)	0.68	44, 135	1.13 (0.74, 1.74)	0.57	44, 135	1.02 (0.63, 1.64)	0.95
*Visit 4*	26, 139	1.08 (0.63, 1.84)	0.79	25, 135	1.29 (0.67, 2.46)	0.44	25, 135	1.20 (0.61, 2.35)	0.59
Combined model (visit 1–3)^*d*^	42, 123			41, 121			41, 121
Visit 1		1.53 (0.97, 2.40)	0.16		1.69 (0.98, 2.92)	0.06		1.62 (0.93, 2.83)	0.09
Visit 2		1.27 (0.81, 2.00)	0.37		1.51 (0.91, 2.50)	0.11		1.43 (0.85, 2.41)	0.18
Visit 3		0.97 (0.64, 1.45)	0.24		0.96 (0.61, 1.52)	0.86		0.85 (0.52, 1.39)	0.52
Male
Geometric average (visit 1–3)	74, 194	0.94 (0.56, 1.58)	0.82	72, 181	0.89 (0.47, 1.70)	0.73	72, 181	0.89 (0.46, 1.70)	0.71
Visit 1	74, 193	1.03 (0.72, 1.46)	0.88	72, 180	0.90 (0.60, 1.34)	0.60	71, 180	0.93 (0.62, 1.39)	0.71
Visit 2	68, 166	1.07 (0.72, 1.59)	0.74	66, 157	1.05 (0.65, 1.71)	0.84	66, 157	1.06 (0.65, 1.74)	0.81
Visit 3	66, 161	0.84 (0.59, 1.21)	0.35	64, 152	0.82 (0.54, 1.26)	0.37	64, 152	0.78 (0.51, 1.21)	0.27
Visit 4	40, 175	1.10 (0.72, 1.68)	0.67	39, 163	1.28 (0.76, 2.18)	0.35	39, 163	1.36 (0.78, 2.36)	0.28
Combined model (visit 1–3)^*d*^	60, 140			58, 133			58, 133
Visit 1		1.09 (0.71, 1.67)	0.69		0.92 (0.57, 1.50)	0.74		0.92 (0.56, 1.50)	0.73
Visit 2		1.13 (0.72, 1.78)	0.59		1.05 (0.60, 1.83)	0.86		1.07 (0.61, 1.88)	0.81
Visit 3		0.83 (0.55, 1.24)	0.36		0.77 (0.48, 1.24)	0.28		0.78 (0.48, 1.26)	0.30
^***a***^Adjusted for specific gravity. ^***b***^Model adjusted for specific gravity, maternal age, race, maternal educational level attained, medical insurance, parity, prior history of PTB, and BMI. ^***c***^Additional adjustment for geometric average sum of DEHP metabolites. ^***d***^Combined model adjusted for all three study visits simultaneously.									

## Discussion

Within our nested case–control study of mothers giving birth in the Boston area, we found no significant associations between averaged or cross-sectional urinary BPA levels and PTB when treating PTB as a single, homogeneous outcome. After further classifying PTB by either spontaneous or placental etiology, no significant relationships with BPA exposure developed, except for significantly elevated odds of spontaneous preterm birth in association with levels measured at visit 4 [median, 35.1 weeks (range, 33.1–38.3 weeks)]. However, these associations should be interpreted cautiously because of the low number of cases in each subgroup. After additional stratification on infant sex, we observed significantly elevated odds of overall PTB for female but not male infants in association with averaged BPA exposure. As far as we are aware, this is the first study to use a more clinical and biological classification of PTB, and to assess BPA exposure at multiple time points during pregnancy with regards to the risk of PTB.

Only one other study has assessed the odds of delivering a PTB in relation to BPA exposure. In a small nested case–control study (*n* = 30 cases, *n* = 30 controls) of PTB in Mexico City, the unadjusted OR of delivering at < 37 weeks in relation to specific gravity–corrected third-trimester urinary BPA concentration was 2.5 (95% CI: 1.1, 6.0) (Cantonwine et al. 2010). It is interesting to note that although the overall results from the present study differ from the one conducted in Mexico City, our analysis from late-pregnancy (visit 4) BPA concentrations with regard to spontaneous PTB closely parallels what was found by Cantonwine et al. (2010). Although type of preterm delivery was not reported by the study authors, the timing of BPA measurements between our study [median, 35.1 weeks (range, 33.1–38.3 weeks)] and the Mexico City study [median, 33.4 weeks (range, 30.6–37.7 weeks)] was very similar. However, only 66 of our 130 cases provided a urine sample at visit 4, and the final gestational age for those cases ranged from 35.0 to 36.9 weeks, reflecting only late preterm births.

Other studies have found inconsistent evidence between BPA and gestational length. In a small study of 40 pregnant women living in southeastern Michigan, there were no differences in gestational length between women with plasma BPA concentrations > 5 and ≤ 5 ng/mL (Padmanabhan et al. 2008). Wolff et al. (2008) found no significant associations between urinary BPA concentration during the third trimester and gestational length among 367 infants living in New York City (NY, USA). Conversely, Weinberger et al. (2014) reported a 1.1-day decrease (95% CI: –2.0, –0.1) in gestational length associated with an inter-quartile range (180.1 ng/mL) increase in BPA urinary concentrations exposure during pregnancy among 72 women living in New Jersey. Reasons for the conflicting evidence between BPA exposure and either risk of PTB or gestational length may include differences in study size and design, differences in populations, use of differing biological media for exposure assessment, or other factors.

In our secondary analysis, we further analyzed preterm birth by differing clinical presentation. Our intention was not only to explore potential mechanistic links between BPA exposure and PTB but also to address potential bias that could arise from including medically indicated protocol-driven PTBs. These preterm births, usually after 36 weeks, are commonly delivered for reasons associated with obstetrical practice rather than actual underlying pathobiology, and without intervention they would have likely proceeded to term. By excluding certain types of protocol-driven PTBs in our data set, we observed a strengthening of our overall associations (i.e., spontaneous + placental PTBs; see Supplemental Material, Table S2), suggesting that inclusion of these PTBs biases our results toward the null.

Placental preterm birth comprised preterm birth following preeclampsia or intrauterine growth restriction (IUGR), which can result from impaired placentation early in pregnancy. The intrauterine environment in early stages of placentation is highly sensitive, and increases in oxidative stress can lead to apoptosis and altered cytotrophoblast turnover rate in the developing placenta (Burton et al. 2009; Heazell and Crocker 2008). Mechanistic evidence exists for the role of BPA in inducing oxidative stress and effecting trophoblast turnover (Babu et al. 2013; Benachour and Aris 2009; Jin and Audus 2005; Morice et al. 2011; Tachibana et al. 2007; Yang et al. 2009). Relevant to placental development, Benachour and Aris (2009) reported dose-dependent apoptosis in isolated primary cytotrophoblast cells from term placentas. Moreover, Morice et al. (2011) demonstrated *in vitro* that BPA exposure at environmentally relevant concentrations can affect the proliferative process in trophoblastic cells through the ERRγ1. Additionally, that study demonstrated the presence of ERRγ1 in several trophoblastic cell lines and isolated extravillous and villous cytotrophoblasts from first-trimester placenta, further suggesting another potential mechanistic link to disruption of normal placental development by exposure to BPA (Morice et al. 2011). Even though our study found no significant relationships between BPA exposure and risk of placental PTB, these results should be interpreted cautiously because we had very few (*n* = 35) placental preterm births in our population, and calculated odds ratios, though not statistically significant, were somewhat elevated.

Spontaneous preterm birth was clinically defined as resulting from spontaneous preterm labor or preterm premature rupture of membranes, both of which primarily arise from inappropriate initiation of an intrauterine inflammatory cascade (Challis et al. 2009). BPA has been shown to stimulate the production of pro-inflammatory cytokines (Lee et al. 2003; O’Brien et al. 2014; Tian et al. 2003) and can induce T-helper (Th)–1 type cytokines while simultaneously suppressing Th-2 cytokines (Youn et al. 2002). Additionally, it has been shown in human populations that BPA concentrations are associated with increased serum C-reactive protein levels (Lang et al. 2008; Yang et al. 2009). It is interesting to speculate that our finding that BPA concentrations measured late in pregnancy (mean gestational age, 35 ± 1 weeks) were associated with significantly increased odds of delivering a spontaneous PTB may be representing an enhanced maternal response to inflammation via BPA exposure leading to either PPROM or spontaneous preterm labor. However, these results should also be interpreted cautiously due to limited numbers of preterm subtypes.

Our results suggest that female infants may be more sensitive to being delivered preterm in relation to gestational BPA exposure than males. Endocrine-disrupting effects of BPA are well studied, and multiple animal and human studies have reported evidence of sex-specific adverse health effects resulting from BPA exposure (Braun et al. 2011b; Kubo et al. 2003; Perera et al. 2012; Rubin et al. 2006). Only one other study has looked at sex-specific relationships with association with BPA exposure and gestational length, but not risk of PTB (Weinberger et al. 2014). Past research has demonstrated that female fetuses are more sensitive to the changes in inflammatory stressors (Cankar et al. 2004; Clifton and Murphy 2004), though further research is needed to understand the potential mechanisms of our findings.

Our study had several strengths, including a repeated time point assessment of BPA exposure, ultrasound dating of gestational age, physician-validated clinical outcomes, and a large number of subjects and preterm cases, which allowed for exploring the heterogeneous nature of PTB. Still, results from our secondary analyses of subtypes of PTB and stratification on infant sex should be interpreted cautiously, given that we were limited in our number of placental and spontaneous PTB cases and are likely to be underpowered to detect subtle relationships. There was also no control for multiple comparisons, which may lead to an inflated type 1 error rate. We acknowledge that the few significant associations found in our analysis may be attributable to chance alone, and larger follow-up studies to replicate the findings are warranted. We substituted BPA values below the LOD with LOD divided by the square root of two in the present study. Although several authors have shown that using a maximum-likelihood estimate for LOD can reduce the inherent bias in using a substitution method for LOD, the improvements are minimal (1–2% reduction in bias) (Cole et al. 2009; Dinse et al. 2014; Guo et al. 2010).

Additionally, this study was limited in our understanding of other residual confounders, such as diet, which may be associated with BPA levels and PTB. It is worth noting that the generalizability of our population may be restricted because Brigham and Women’s Hospital is a major referral center for high-risk pregnancies; thus, our population might have a higher than normal proportion of women with prior conditions that could lead to medically indicated protocol-driven PTB (e.g., placement of abdominal cerclage for cervical insufficiency). Regardless, this population was originally collected in a prospective fashion from a major regional hospital in a large urban center whose overall demographic breakdown of delivery patients is reflective of the general U.S. population. Our choice of a nested case–control design stemmed from financial considerations for exposure assessment and may have some inherent limitations. Analyzing continuous gestational age in a time-to-event framework in the entire cohort would yield more informative inference if we could obtain exposure measures on a larger cohort; however, given the resources, the case–control design maximizes our power to detect exposure–outcome associations. Although, to our knowledge, this was the first study of preterm birth or gestation length to use multiple urinary BPA concentrations from each woman, the low temporal reliability of BPA concentrations across pregnancy may indicate that even with three to four repeated measures, there may still be substantial nondifferential exposure measurement error, which would further limit statistical power to detect associations.

## Conclusions

In conclusion, although we found no consistently significant associations between urinary BPA concentrations and prematurity, further research may be warranted given our significant adverse findings with spontaneous PTB and late-pregnancy urinary BPA concentrations and stratified analysis effects in females. Additionally, this study highlights the need to model PTB by underlying causes in population studies not only to help inform potential mechanistic links, but also to reduce potential bias from inclusion of medically indicated protocol driven PTBs.

## Supplemental Material

(201 KB) PDF
