# Supplementary material for: Urinary Bisphenol A Levels during Pregnancy and Risk of Preterm Birth
Source: Environ Health Perspect. 2015 Mar 27;123(9):895–901. doi: 10.1289/ehp.1408126 (PMC4559950; doi:10.1289/ehp.1408126)
Supplement: (201 KB) PDF [file ehp.1408126.s001.acco.pdf]

Environ Health Perspect
doi: 10.1289/ehp.1408126

Note to Readers: ***EHP*** **strives to ensure that all journal content is accessible to all readers. However, some figures and Supplemental Material published in** ***EHP*** **articles may not conform to 508 standards due to the complexity of the information being presented. If you need assistance accessing journal content, please contact ehp508@niehs.nih.gov. Our staff will work with you to assess and meet your accessibility needs within 3 working days.**

Supplemental Material

Urinary Bisphenol A Levels during Pregnancy and Risk of Preterm Birth

David E. Cantonwine, Kelly K. Ferguson, Bhramar Mukherjee, Thomas F. McElrath, and John D. Meeker

Table of Contents

Table S1. Odds ratios (95% confidence intervals) of overall preterm birth in association with quartiles of random BPA slopes and intercepts generated by linear mixed effect models.

Table S2. Odds ratios (95% confidence intervals) of combined preterm sub-categories in association with ln-unit increase in BPA concentration (ng/mL).

Table S1. Odds ratios (95% confidence intervals) of overall preterm birth in association with quartiles of random BPA slopes and intercepts generated by linear mixed effect models.

	**Model 1**			**Model 2[a]**			**Model 3[b]**		
**Overall Preterm**	**N (cases, controls)**	**OR (95% CI)**	**p**	**N (cases, controls)**	**OR (95% CI)**	**p**	**N (cases, controls)**	**OR (95% CI)**	**p**
Random Intercept	130, 352	1.64 (0.73, 3.65)	0.23	127, 334	1.56 (0.61, 4.01)	0.36	127, 334	1.43 (0.55, 3.70)	0.46
Quartiled slopes of BPA exposure across pregnancy									
Quartile 1 < –0.000199		REF			REF			REF	
Quartile 2 –0.000199 to –0.0000164		0.60 (0.33, 1.07)	0.09		0.55 (0.29, 1.05)	0.07		0.54 (0.28, 1.03)	0.06
Quartile 3 –0.0000165 to 0.000174		1.01 (0.59, 1.75)	0.97		0.94 (0.52, 1.72)	0.84		0.88 (0.48, 1.63)	0.68
Quartile 4 ≥0.000175		0.78 (0.45, 1.37)	0.39		0.81 (0.43, 1.53)	0.51		0.76 (0.40, 1.45)	0.41

[a]Model adjusted for specific gravity, maternal age, race, maternal educational level attained, medical insurance, parity, prior history of PTB, and BMI.

[b]Additional adjustment for geometric average sum of DEHP metabolites.

Table S2. Odds ratios (95% confidence intervals) of combined preterm sub-categories in association with ln-unit increase in BPA concentration (ng/mL).

	Model 1[a]			Model 2[b]			Model 3[c]		
**Spontaneous/Placental PTB**	**N (cases, controls)**	**OR (95% CI)**	**p**	**N (cases, controls)**	**OR (95% CI)**	**p**	**N (cases, controls)**	**OR (95% CI)**	**p**
GM Average (Visit 1-3)	91, 352	1.52 (1.02, 2.27)	0.04	89, 338	1.45 (0.89, 2.37)	0.13	89, 338	1.33 (0.81, 2.18)	0.27
Visit 1	91, 351	1.27 (0.96, 1.67)	0.09	89, 337	1.10 (0.81, 1.51)	0.54	89, 337	1.05 (0.76, 1.45)	0.75
Visit 2	83, 304	1.28 (0.95, 1.74)	0.11	81, 295	1.35 (0.95, 1.90)	0.09	81, 295	1.34 (0.94, 1.91)	0.10
Visit 3	79, 301	0.95 (0.70, 1.27)	0.70	77, 291	0.91 (0.65, 1.28)	0.59	77, 291	0.81 (0.56, 1.18)	0.27
Visit 4	40, 314	1.51 (1.02, 2.22)	0.04	38, 302	1.96 (1.23, 3.12)	0.004	38, 302	1.80 (1.11, 2.92)	0.02
Combined Model (Visit 1-3)[d]	73, 263			71, 256			71, 256		
Visit1		1.18 (0.83, 1.68)	0.36		1.06 (0.72, 1.58)	0.76		1.05 (0.70, 1.56)	0.82
Visit2		1.30 (0.92, 1.83)	0.14		1.43 (0.97, 2.11)	0.07		1.39 (0.93, 2.06)	0.11
Visit3		0.89 (0.65, 1.23)	0.48		0.84 (0.59, 1.20)	0.33		0.79 (0.55, 1.14)	0.21

[a]Adjusted for specific gravity. [b]Model adjusted for specific gravity, maternal age, race, maternal educational level attained, medical insurance, parity, prior history of PTB, and BMI. [c]Additional adjustment for geometric average sum of DEHP metabolites. [d]Combined model adjusted for all three study visits simultaneously.
